# α-Hemolysin-Aided Oligomerization of the Spike Protein RBD Resulted in Improved Immunogenicity and Neutralization Against SARS-CoV-2 Variants

**DOI:** 10.3389/fimmu.2021.757691

**Published:** 2021-09-24

**Authors:** Jintao Zou, Haiming Jing, Xiaoli Zhang, Yiheng Liu, Zhuo Zhao, Lianli Duan, Yue Yuan, Zhifu Chen, Qiang Gou, Qingshan Xiong, Sisi Li, Feng Yang, Hao Zeng, Quanming Zou, Jinyong Zhang

**Affiliations:** ^1^ National Engineering Research Center of Immunological Products, Department of Microbiology and Biochemical Pharmacy, College of Pharmacy, Third Military Medical University, Chongqing, China; ^2^ Department of Clinical Hematology, College of Pharmacy, Third Military Medical University, Chongqing, China

**Keywords:** SARS-CoV-2, COVID-19, subunit vaccine, mHla-RBD, oligomerization

## Abstract

The increase in confirmed COVID-19 cases and SARS-CoV-2 variants calls for the development of safe and broad cross-protective vaccines. The RBD of the spike protein was considered to be a safe and effective candidate antigen. However, the low immunogenicity limited its application in vaccine development. Herein, we designed and obtained an RBD heptamer (mHla-RBD) based on a carrier protein-aided assembly strategy. The molecular weight of mHla-RBD is up to 450 kDa, approximately 10 times higher than that of the RBD monomer. When formulated with alum adjuvant, mHla-RBD immunization significantly increased the immunogenicity of RBD, as indicated by increased titers of RBD-specific antibodies, neutralizing antibodies, Th2 cellular immune response, and pseudovirus neutralization activity, when compared to RBD monomer. Furthermore, we confirmed that RBD-specific antibodies predominantly target conformational epitopes, which was approximately 200 times that targeting linear epitopes. Finally, a pseudovirus neutralization assay revealed that neutralizing antibodies induced by mHla-RBD against different SARS-CoV-2 variants were comparable to those against the wild-type virus and showed broad-spectrum neutralizing activity toward different SARS-CoV-2 variants. Our results demonstrated that mHla-RBD is a promising candidate antigen for development of SARS-CoV-2 vaccines and the mHla could serve as a universal carrier protein for antigen design.

## Introduction

Severe acute respiratory syndrome coronavirus 2 (SARS-CoV-2) has brought about a rapidly spreading pandemic of coronavirus disease 2019 (COVID-19) since December 2019 (1, 2). More than 192 million cases were confirmed, with 4.1 million deaths worldwide by July 21, 2021. Vaccines are considered to be the most cost-effective means to combat infectious diseases. To date, more than 260 SARS-CoV-2 vaccines are under development at different stages, and 10 of them have been approved for clinical administration (3–5), which plays essential roles in halting the pandemic and its destructive effects on global public health and the economy.

In addition to inactivated vaccines, almost all other SARS-CoV-2 vaccines use full-length or truncated spike (S) protein as antigens due to its critical biological significance in the pathogenesis of the virus (6). The S protein is one of the major surface exposure glycoproteins of SARS-CoV-2. It consists of 1273 amino acids and contains two subunits, S1 and S2 (7). The S1 subunit facilitates the binding of the virus to the ACE2 receptor on the host cell membrane, whereas the S2 subunit participates in viral fusion, which involves priming of the S protein by proteases on the cell surface and subsequent entry and replication of the virus in the host cell cytoplasm (8). The receptor-binding domain (RBD), responsible for recognizing the ACE2 receptor on host cells, is located on the C-terminus of the S1 subunit (9). Several studies have revealed that the RBD contains the most immune-dominant neutralizing epitopes. Nearly 90% of the neutralizing antibodies in the serum of convalescent patients are induced by the RBD (10). Thus, the RBD is considered as the most promising candidate antigen for SARS-CoV-2 vaccine development.

However, RBD is a small protein with a molecular weight of approximately 32 kDa, and it exists as a monomer in solution (11); thus, immunogenicity is limited. The titer of neutralizing antibodies induced by RBD, which is closely related to the severity of the disease and can effectively block the replication of the virus and eliminate the virus in the patient, is even lower (12). Thus, strategies should be introduced to enhance the immunogenicity of this protein during vaccine development. A modified RBD dimer generated higher neutralizing antibodies than monomeric proteins (13); another study also reported that RBD fused with the Fc domain showed a highly potent SARS-CoV-2 neutralizing antibody response (14). In addition, several studies have confirmed that the use of nanoparticles to carry RBD antigens can also greatly improve the immunogenicity of RBD, and induce higher neutralizing antibodies and T cell responses (15, 16). These studies shed light on improving the immunogenicity of RBD by increasing its size and molecular complexity.

α-Hemolysin (Hla) is a pore-forming toxin secreted by *Staphylococcus aureus* that can self-assemble into a heptameric pore structure on host cell membranes, resulting in cell lysis and death (17). A previous study revealed that an Hla mutant (mHla) lacking the two β-sheets that form the pore-like structure retains the ability to assemble into a heptamer but loses its biological activity (18). We have demonstrated that mHla could serve as a carrier protein to aid the assembly of IC43, a candidate antigen of *Pseudomonas aeruginosa* (19), resulting in improved immunogenicity and protective efficacy of the latter. The mechanism mainly lies in two aspects. First, the formation of oligomers increases the size of IC43. Second, fusion with mHla promotes the exposure of hidden epitopes on IC43 (20).

In this study, to validate whether mHla fusion could be used as a universal strategy in antigen design, we constructed a mHla-RBD fusion protein. We confirmed that this protein exists as a heptamer solution. Using the monomer of RBD as a control, our results showed that immunization with mHla-RBD induced an improved SARS-CoV-2 neutralizing antibody response in BALB/c mice, and antisera from mHla-RBD-immunized mice showed broad neutralizing activities toward SARS-CoV-2 variants. Thus, this fusion protein is a promising and novel candidate for further SARS-CoV-2 vaccine development.

## Materials And Methods

### Pseudovirus and Cell Lines

All SARS-CoV-2 sipke pseudoviruses that use GFP as a reporter gene, including the wild type, alpha variant, beta variant, and gamma variant, were purchased from Packgene Company. The titers were measured by Packgene. All pseudovirus assays were performed in laboratories with biosafety level 2. 293T cell lines were infected with human angiotensin-converting enzyme 2 (ACE2) lentiviruses, and stable cell lines (hACE2-293T) were established.

### DNA Manipulation

The coding sequence for the RBD region, which spans residues 319-529 of the S protein of the SARS-CoV-2 Wuhan-Hu-1 isolate (accession number MN908947), and the coding sequence for *S. aureus* Hla (accession number AP017922.1) were obtained from PubMed. The sequence encoding the signal peptide (residues 1-26) of Hla was removed. The sequence encoding the two β sheets involved in forming the heptameric pore was replaced by a sequence encoding “PSGS”, as reported previously (18). Then, the two sequences were linked together by a “GGGGS” linker, with sequences encoding an N-terminal TPA signal peptide and a C-terminal 8×His tag. The resultant sequence was codon optimized for human cells, synthesized and inserted into the eukaryotic expression vector pcDNA3.1 (Novagen, Madison, WI, USA) *via BamH1* and *XhoI* restriction sites by GeneCreate Biological Engineering Co., Ltd. (Wuhan, Hubei Province, China). This resulted in the recombinant plasmid pcDNA3.1-mHla-RBD. The plasmid pcDNA3.1-RBD was constructed under the same protocol as described for RBD.

### Protein Expression and Purification

HEK-293F cells (Thermo Fisher) were transiently transfected using PEI 25K (Polysciences) with plasmids pcDNA3.1-mHla-RBD or pcDNA3.1-RBD. After 5 days in shaker culture, media were collected and cleared of debris for 20 min by centrifugation at 6,000 g and filtered using 0.45-mm flasks (Millipore). Proteins in media were loaded onto Ni Sepharose Excel beads (GE Healthcare, Piscataway, NJ, USA), washed with 20 mM phosphate buffer pH 8.0, 300 mM NaCl and 20 mM imidazole, and eluted with 20 mM phosphate buffer pH 8.0, 300 mM NaCl and 300 mM imidazole. Eluates were buffer exchanged with phosphate-buffered saline (PBS) using a HiPrep™ 26/10 desalting column (GE Healthcare, Piscataway, NJ, USA). The peak fractions corresponding to the recombinant proteins were pooled and verified by SDS-PAGE, and the concentration was determined using the BCA method. All purified proteins were stored at -80°C before use.

### Oligomeric State Evaluation

The oligomeric state of mHla-RBD was determined by two methods. For chemical cross-linking analysis, mHla-RBD was incubated with 0.01%, 0.05%, 0.1%, and 0.2% glutaraldehyde at 37°C for 30 min. The cross-linking reaction was then terminated by adding a loading buffer containing SDS and glycine, and the protein samples were then analyzed by SDS-PAGE. For gel filtration analysis, 200 μl purified mHla-RBD was loaded onto the Superdex™ 200 10/300 GL column. The elution volume of the corresponding peak was used to calculate the molecular weight, determining the oligomeric state of the protein.

### Hemolytic Activity Assay

The hemolytic activity assay was carried out according to a method established by us previously (21). In brief, 100μl of rabbit erythrocyte suspension in PBS (4%) was mixed with 100 μl of serial diluted wild type Hla or mHla-RBD (256 - 0.125μg/ml), an equal volume of 1% Triton X-100 and PBS were used as positive and negative controls, respectively. After a 30 min incubation at 37°C, the mixtures were centrifuged at 400 × g for 10 min. The hemolytic activity was determined by the release of hemoglobin, measured spectrophotometrically at 540 nm and presented as % hemolysis of the positive control (Triton X-100).

### Immunization

Six- to eight-week-old female BALB/c mice were purchased from Beijing HFK Bioscience Limited Company (Beijing, China) and kept under specific pathogen-free (SPF) conditions during the experiment. mHla-RBD and RBD were diluted with PBS and formulated with Al(OH)_3_ adjuvant (Pierce) at a ratio of 1:1 (v:v). Mice (n=10) were immunized intramuscularly into quadriceps muscle with 100 µl of the mixture containing 20 µg RBD or 10, 20, and 40 µg of mHla-RBD on days 0, 14, and 28. Negative control mice were immunized with an equal volume of PBS plus adjuvant.

### ELISA

One week after each boost immunization (days 21 and 35), mice were exsanguinated, and serum samples were collected for the enzyme-linked immunosorbent assay (ELISA) of RBD-specific antibodies. Wells of microtiter plates (Thermo Labsystems) were coated with RBD (2 µg per well) in 50 mM carbonate buffer (pH 9.5) overnight at 4°C. Serum samples were serially diluted 2-fold in PBS (starting at 1:1000) and used as the primary antibodies. The secondary antibodies were HRP-conjugated goat anti-mouse IgG, IgG1 or IgG2a (Sigma). Absorbance was read at 450 nm (OD450), and the titers were defined as the highest dilution that yielded an absorbance value of more than twice the value of the preimmune serum. According to the manufacturer’s protocol, the titer of neutralizing antibody was detected using the Anti-SARS-CoV-2 Neutralizing Antibody Titer Serologic Assay Kit (Acro Biosystems, Beijing, China).

### ELISPOT

ELISPOT assays were carried out to detect interferon γ (IFN-γ) and IL-4 released by splenocytes isolated from immunized mice as described previously (22). Splenocytes from immunized mice were stimulated with RBD (50 μg/ml) for 60 hours. Detection was performed according to the manufacturer’s instructions (BD, USA).

### Peptide Synthesis and Immune-Dominant Linear Epitope Mapping

Thirty-five synthetic overlapping peptides, which cover the entire length of the RBD, were synthesized by China Peptides Co., Ltd. Each peptide consisted of 18 amino acid residues, with an overlap of 12 amino acids each. The purity of these peptides was 90% or higher. The peptides were dissolved in dimethyl sulfoxide at a concentration of 0.5 mg/mL and stored at -80°C before use. Immune-dominant epitope mapping was carried out by ELISA. In brief, wells of microtiter plates were coated with each peptide diluted in hydrogen bicarbonate buffer (pH 9.6) at a concentration of 5 mM. Serum samples from immunized mice were diluted 500-fold with PBS and used as the primary antibodies. The secondary antibody was HRP-conjugated goat anti-mouse IgG (Sigma). The ELISA results were given as absorbance values at 450 nm.

### Detection of RBD-Specific Antibodies Targeting Conformational Epitopes

To detect RBD-specific antibodies targeting conformational epitopes, we followed a previously reported protocol with minor adjustments (23). The linear peptide pool (20 µg/ml) was coated on 96-well ELISA plates, sera from immunized mice (1:100,000 dilution), and were then added and incubated 3 times to absorb linear epitope-recognizing antibodies. Each incubation required 1 hour. After the last incubation, the supernatant was collected, pooled, and used in ELISAs with 96-well ELISA plates coated with a linear peptide pool (2 µg/ml) and RBD (2 µg/ml). ELISA was performed as described above.

### Pseudovirus Neutralization Assay *via* High Content Analysis

Pseudovirus neutralization was carried out following protocols described previously (24). In brief, hACE2-293T cells were seeded into 384-well plates (5x10^3^ per well) and grown overnight. Twofold serially diluted heat-inactivated serum samples in containing 2% fetal bovine serum (FBS) high-glucose DMEM (Gibco) were incubated with 100 TCID_50_ of pseudovirus-GFP for 30 min at 37°C. The mixtures were transferred to 384-well plates (Cellvis). After incubation for 24 hours, 2% FBS high-glucose DMEM was replaced with 10% FBS high-glucose DMEM to allow sufficient GFP expression for the next 48 hours. Finally, high content analysis was performed on Opera Phenix (PerkinElmer). Each well was imaged and calculated with an average fluorescence intensity of 488 nm (AFI488). The inhibition percentage of each well was calculated with the formula inhibition (%) = (1-(AFI488_assay_-AFI488_blank control_)/(AFI488_infection control_- AFI488_blank control_))×100%. The 50% inhibition titer (IT_50_) was calculated with a dose-response inhibition (normalized response) model by GraphPad Prism 8.0.

### Statistical Analysis

Data are presented as the means ± standard deviation (SD), means ± 95% confidence interval (CI), or means ± standard error of the mean (SEM). The means were compared using the two-tailed Student’s t-test and one-way ANOVA with Tukey’s multiple comparisons. The analyses were performed using GraphPad Prism 8.0 (GraphPad Software), and P<0.05 was considered statistically significant, and the protein structures were visualized by PyMOL.

## Results

### Design, Preparation, and Characterization of the mHla-RBD Heptamer

**Figure 1:**
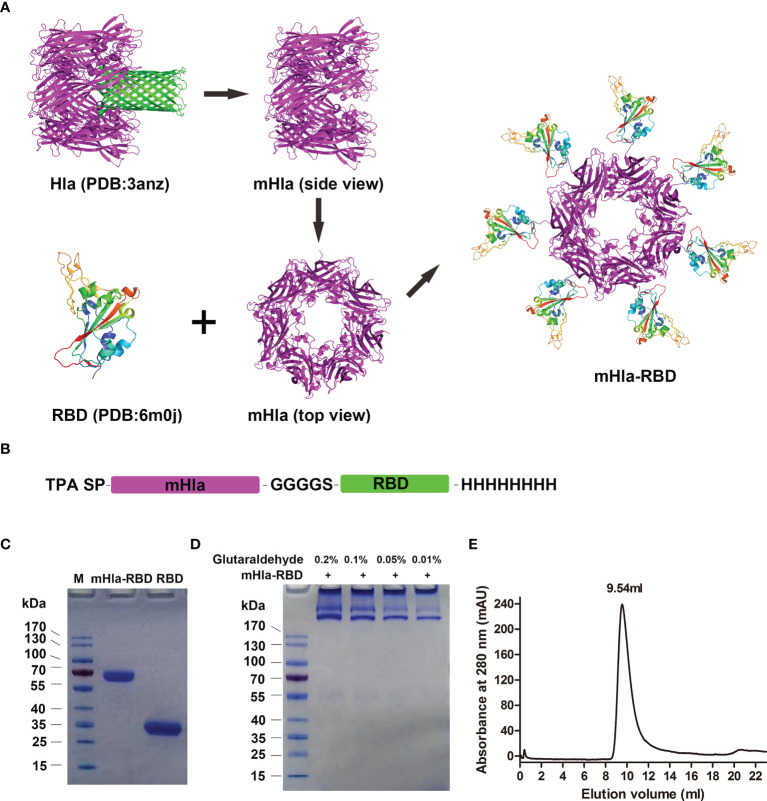
Design, preparation, and characterization of mHla-RBD. **(A)** The schematic diagram of RBD heptamer design. mHla, which lucks the transmembrane β-barrel structure, was used as the carrier protein, and RBD was located on the surface of mHla. **(B)** The designation of the fusion protein. **(C)** SDS-PAGE analysis of purified RBD and mHla-RBD. **(D)** SDS-PAGE analysis of mHla-RBD after treatment with 0.01%, 0.05%, 0.1%, and 0.2% glutaraldehyde. **(E)** Oligomeric state of mHla-RBD determined by size-elution chromatography.

To obtain the haptamer of RBD, we introduced a carrier protein-aided assembly strategy as described previously (20). mHla was used as a carrier protein, and RBD was displayed on the surface of the carrier protein, similar to that of “phage display” (**Figure 1A**). Structural analysis indicated that the C-terminus of Hla is exposed at the surface (25); thus, the RBD is placed at the C-terminus of the fusion protein. In addition, a flexible linker, GGGGS, was placed between mHla and RBD, as the amino acids in this linker do not contain any side chains, so it does not play much a role in protein secondary structure formation (26). The TPA signal peptide (SP) was placed at the N-terminus of the fusion protein to ensure the secretion of the recombinant protein (27), and an 8×His tag was placed at the C-terminus to facilitate purification of the target protein (**Figure 1B**). Both RBD and mHla-RBD were highly expressed in HEK-293F cells, and the purity of these proteins was up to 95%, as determined by SDS-PAGE after affinity chromatography and desalting (**Figure 1C**). The molecular weights were approximately 32 and 65 kDa for RBD and mHla-RBD, respectively.

Next, chemical cross-linking analysis was applied to determine the oligomeric state of mHla-RBD. As shown in **Figure 1D**, mHla-RBD formed oligomers on the gel completely in the presence of glutaraldehyde, even though the concentration was as low as 0.01%, but the acute oligomeric state of mHla-RBD was not clear. We further evaluated the oligomeric state of mHla-RBD by gel filtration analysis. As shown in **Figure 1E**, the elution volume for mHla-RBD was 9.54 ml when applied to the SuperdexTM 200 10/300 GL column, and the elution volume of the protein standard ferritin (440 kDa) was 9.78 ml, this result indicated that mHla-RBD exists as a heptamer in solution. In contrast, RBD was reported previously appears as a monomer in solution (11). In this study, when RBD was applied to gel filtration analysis, the elution volume was 15.84 ml, which clearly indicated that it existed as a monomer in solution (**Supplementary Figure 1**).

Further, hemolytic activity assay showed that wild-type Hla lysis rabbit erythrocyte in a concentration dependent manner, with 50% of hemolysis at 0.6958 μg/ml. In contrast, mHla-RBD didn’t show any hemolysis activity even the concentration reached 256 μg/ml (**Supplementary Figure 2**). This result indicated that mHla-RBD is safe for vaccine development.

### mHla-RBD Immunization Resulted in Improved Immunogenicity Compared to RBD Monomer

**Figure 2:**
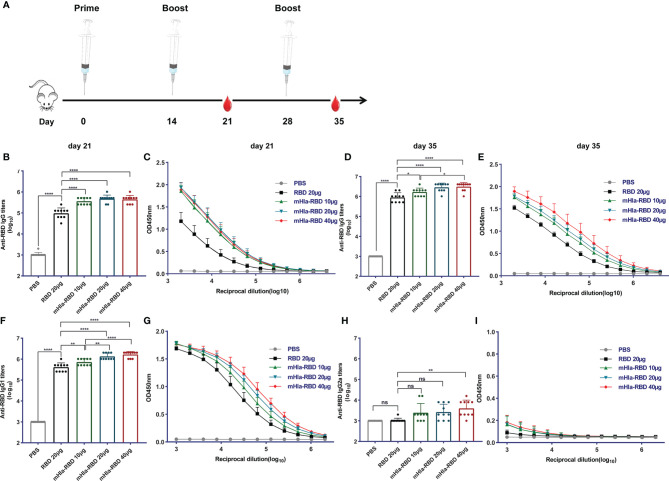
mHla-RBD exhibited improved immunogenicity compared to the RBD monomer. **(A)** The immunization and sampling schematic diagram. BALB/c mice were immunized with PBS, RBD, and different dosages of mHla-RBD with alum adjuvant. One week after the second immunization (day 21), SARS-CoV-2 RBD-specific immunoglobulin gamma (IgG) antibody titers **(B)** and different dilutions of IgG **(C)** were detected by ELISA. One-way ANOVA with Tukey’s multiple comparisons, ****p <0.0001. One week after the third immunization (day 35), SARS-CoV-2 RBD-specific immunoglobulin gamma (IgG) antibody titers **(D)** and different dilutions of IgG **(E)** were detected by ELISA. One-way ANOVA with Tukey’s multiple comparisons, *p <0.05, ****p <0.0001. Assessment of IgG1 antibody titer **(F)** and different dilutions of IgG1 **(G)**, one-way ANOVA with Tukey’s multiple comparisons, **p <0.01, ****p <0.0001. Measurement of IgG2a antibody titer **(H)** and different dilutions of IgG2a **(I)**, one-way ANOVA with Tukey’s multiple comparisons, **p <0.01; ns, not significant.

To evaluate the potential of mHla-RBD as a candidate antigen, BALB/c mice (n=10) were immunized three times with increasing doses of mHla-RBD formulated with alum adjuvant. PBS and RBD plus adjuvant were used as controls. Serum samples were collected 7 days after the second and last immunizations as indicated (**Figure 2A**). After the second immunization, RBD-specific IgG in serum from 10 µg of mHla-RBD-immunized mice reached an endpoint titer of 10^5.5^, which was significantly higher than 20 µg of RBD immunization (lower than 10^5^). Since the molecular weight of mHla is almost the same as that of RBD, this result indicated that 5 µg of RBD heptamer exhibited higher immunogenicity than 20 µg of RBD monomer. Meanwhile, serum from mice immunized with 20 µg of mHla-RBD showed a higher titer of RBD specific IgG when compared to 10 µg of mHla-RBD, but showed no difference when compared with 40 µg of mHla-RBD (**Figures 2B, C**). Furthermore, the titers of RBD-specific IgG from mice after the third immunization were significantly elevated, reached an endpoint titer of ~10^6^ or higher, and showed a similar tendency for each group when compared to the second immunization (**Figures 2D, E**). These results demonstrated that oligomerization of the RBD resulted in improved immunogenicity and that 20 µg of mHla-RBD was sufficient to induce an optimal immune response. Meanwhile, we also determined the titers of anti-Hla antibodies in the serum of immunized mice in each group. The results showed that the titers in mHla-RBD immunized groups were significantly higher than PBS and RBD immunized groups, but showed no different in mice immunized with different dose of mHla-RBD (**Supplementary Figure 3**). Surprisingly, the titers of anti-RBD antibodies were approximately 10 times higher than that of anti-Hla antibodies, which indicated that Hla had little influence on host immune response against RBD.

Next, the levels of RBD-specific IgG1 and IgG2a antibodies were determined to detect a possible Th1- or Th2-biased immune response (28). As shown in **Figures 2F, G**, immunization with 10 µg of mHla-RBD significantly improved the titers of RBD-specific IgG1 antibodies compared to immunization with 20 µg of RBD, titers increased in a dose-dependent manner. In contrast, RBD-specific IgG2a-specific antibodies showed differences only when the dose of mHla-RBD reached 40 µg (**Figures 2H, I**). However, the titers of IgG1 were 100 to 1000 times higher than those of IgG2a. Thus, fusion with mHla mainly induces a Th2 biased immune response.

### Cellular Immune Response Induced by mHla-RBD Immunization

**Figure 3:**
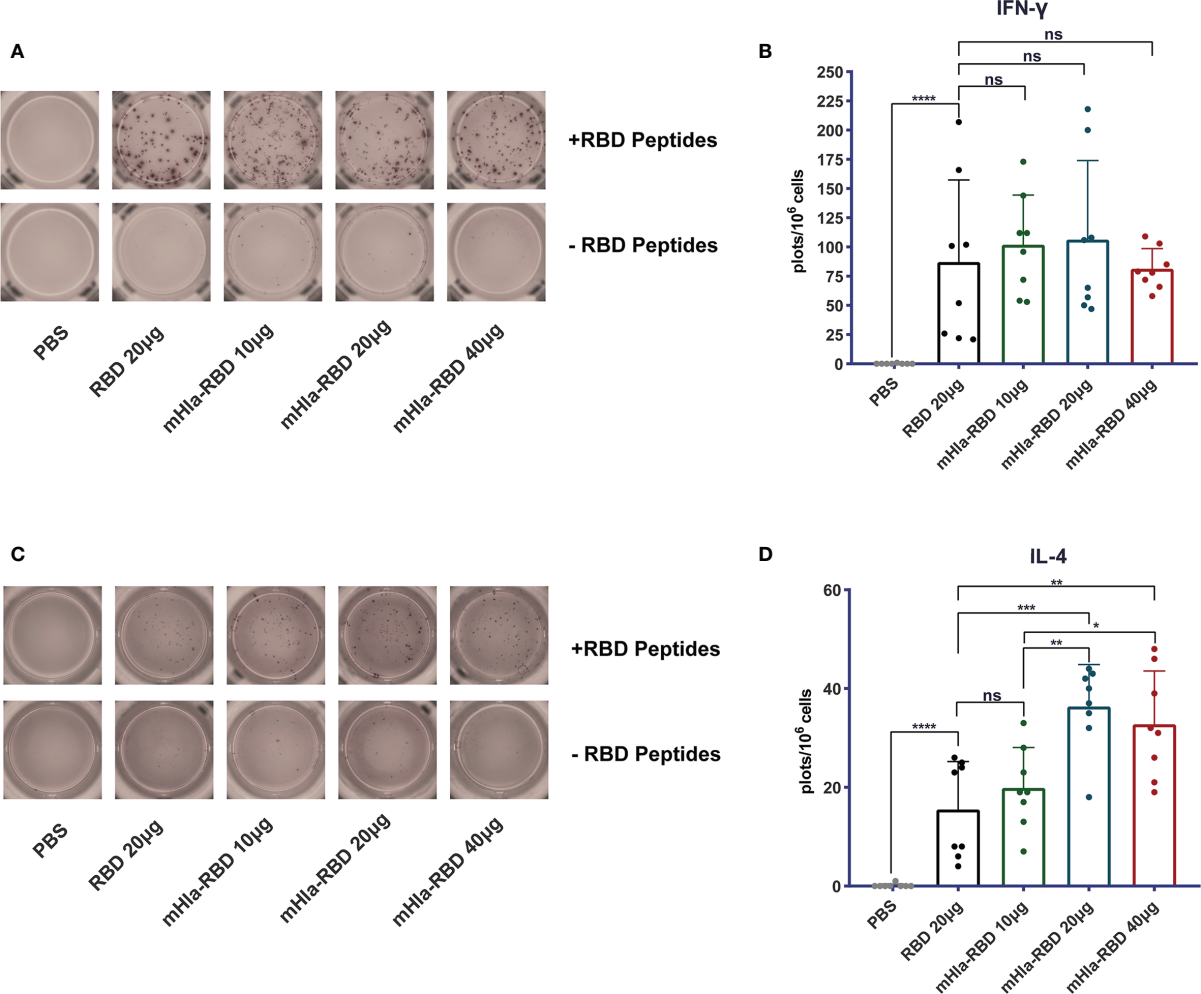
Detect of cellular response induced by RBD and mHla-RBD immunization. ELISpot analysis of IFN-γ **(A)** and IL-4 **(C)** secretion by splenocytes from immunized BALB/c mice. One-way ANOVA with Tukey’s multiple comparisons, *p <0.05, **p <0.01, ***p <0.001, ****p <0.0001; ns, not significant. Representative images of IFN-γ **(B)** and IL-4 **(D)** ELISpot analyses.

Cellular immune responses are essential host defense against SARS-CoV-2 infection. We determined the frequency of IL-4- and IFN-γ-producing cells in splenocytes of mice immunized 3 times with RBD and different doses of mHla-RBD by ELISPOT assay. The results showed that the frequency of IL-4-producing cells was doubled in mice immunized with 20 µg of mHla-RBD compared to the same dose of RBD (**Figures 3C, D**). In contrast, the frequency of IFN-γ-producing cells was increased in the antigen-immunized groups compared to the PBS immunization group but showed no difference among the different antigens (**Figures 3A, B**). These results suggested that mHla fusion enhanced the Th2 but not Th1 cellular immune response, which was consistent with the antibody subtype assay.

### RBD-Specific Antibodies Predominantly Targeting Conformational Epitopes

**Figure 4:**
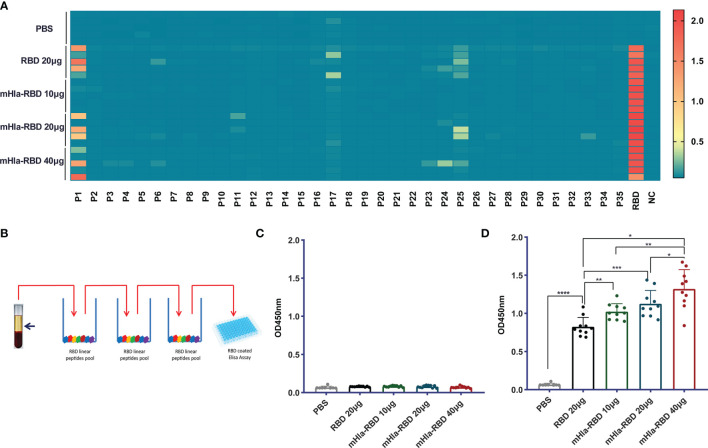
Detect of linear and conformational epitope antibodies. **(A)** Heatmap of antibodies recognizing 18-mer overlapping peptides from immunized BALB/c mice (n=5). **(B)** Schematic diagram of elimination of antibodies recognizing RBD linear epitopes. **(C)** Assessment of antibodies recognizing the RBD linear peptide pool after absorption. **(D)** Assessment of antibodies recognizing RBD conformational epitopes after absorption. One-way ANOVA with Tukey’s multiple comparisons, *p <0.05, **p <0.01, ***p <0.001, ****p <0.0001.

Previously, we showed that mHla efficiently increased the immunogenicity and protective efficacy of IC43, partially by promoting the exposure of hidden epitopes (20). We also mapped the linear B cell epitopes on the RBD using serum from different immunization groups. As shown in **Figure 4A**, three linear peptides, P1, P17, and P25, were positive when mapped with 500-fold diluted serum from RBD-immunized mice. In contrast, no positive peptide was identified with serum from mice immunized with 10 µg of mHla-RBD. When the dose of mHla-RBD increased to 20 µg and 40 µg, P25 tended to be positive, but the OD450 value toward these epitopes was lower than that of RBD-immunized controls. Interestingly, P25 was also reported to contain a linear epitope in another study. Antibodies recognizing this epitope exhibited no virus-neutralizing activity (14), which indicated that linear epitopes in the RBD may not be essential to inducing neutralizing antibodies.

We then established a method to determine the relative titers of antibodies targeting conformational epitopes in the serum of immunized mice (**Figure 4B**). In short, serum samples were diluted 100,000-fold with PBS and loaded onto 96-well plates coated with a peptide pool of RBD. After 3 rounds of absorption, antibodies targeting linear epitopes were removed successfully (**Figure 4C**), and the OD450 value of antibodies targeting conformational epitopes was determined, the level of which was significantly higher in mice immunized with 10 µg of mHla-RBD than in those immunized with 20 µg of RBD monomer, and a higher level was observed with an increased dose of mHla-RBD (**Figure 4D**). Interestingly, although 100,000-fold diluted sera were used in this experiment, the levels of antibodies were comparable to those targeting linear epitopes after 500-fold dilution. This result indicated that the amount of antibodies recognizing conformational epitopes is predominant in immunized serum, consistent with a form report that conformational epitopes occupy more than 90% of all epitopes within an antigen (29).

### Immunization With mHla-RBD Induced a More Potent Neutralizing Response Than RBD Monomer

**Figure 5:**
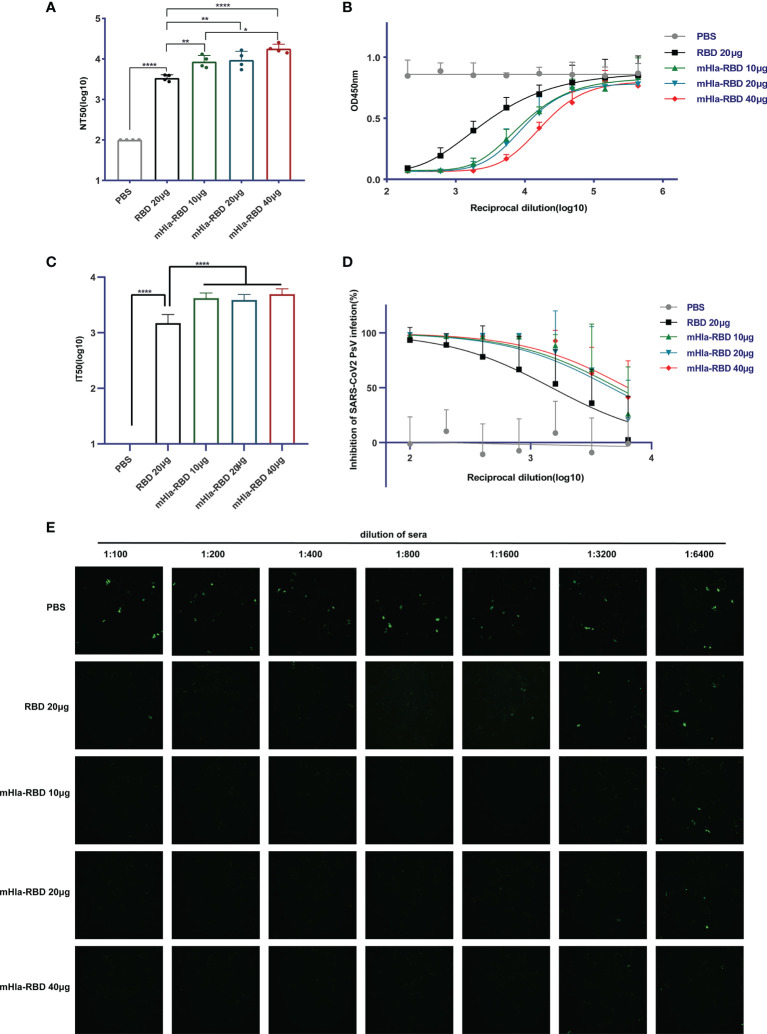
Neutralizing antibodies assay. **(A)** ELISA blocking assay. One-way ANOVA with Tukey’s multiple comparisons, *p <0.05, **p <0.01, ****p <0.0001. **(B)** Relative amount of RBD-HRP binding to hACE2 after blocking sera from immunized BALB/c mice with different dilutions. **(C)** Wild-type-spike pseudovirus neutralization assay. Two-tailed unpaired Student’s t-test, ****p <0.0001. **(D)** Inhibition of wild-type-spike pseudovirus infection by sera from immunized BALB/c mice. **(E)** Presentative images of wild-type-spike pseudovirus infection blocked by serial dilution of sera from immunized BALB/c mice.

We then determined the titers of neutralizing antibodies in the serum of mice from different immunization groups. As expected, immunization with 10 µg of mHla-RBD induced NT_50_ up to 1:8573 after three injections, significantly higher than that induced by 20 µg of RBD immunization (1:3412). Furthermore, the NT_50_ of serum from mHla-RBD-immunized mice increased in a dose-dependent manner, reaching 1:17906 when 40 µg of mHla-RBD was applied (**Figure 5A**). The relative amount of RBD-HRP binding to hACE2 after blocking is presented as the absorbance value at 450 nm (**Figure 5B**). Furthermore, a pseudovirus neutralization assay was performed. The serum of mice immunized with PBS could not inhibit wild-type-spike pseudovirus infection. The IT50 of serum immunized with 20 µg of RBD was 1:1496. In contrast, the IT50 of serum immunized with 20 µg of mHla-RBD increased to approximately 1:4000 (**Figure 5C**). The normalized inhibition percentage of serially diluted serum depicted a different trend between the RBD and mHla-RBD groups; similarly, stronger inhibition was observed in the mHla-RBD groups than in the RBD group (**Figure 5D**). The difference was clearly detected by the bare eye, which showed the difference in the inhibition of pseudovirus infection by serum from mice immunized with different antigens (**Figure 5E**). Together, these results revealed that immunization with mHla-RBD induced significantly stronger neutralization against wild-type RBD or wild-type spike pseudovirus than immunization with RBD.

### mHla-RBD Immunization Induced Cross-Protective Efficacy Against SARS-CoV-2 Variants

**Figure 6:**
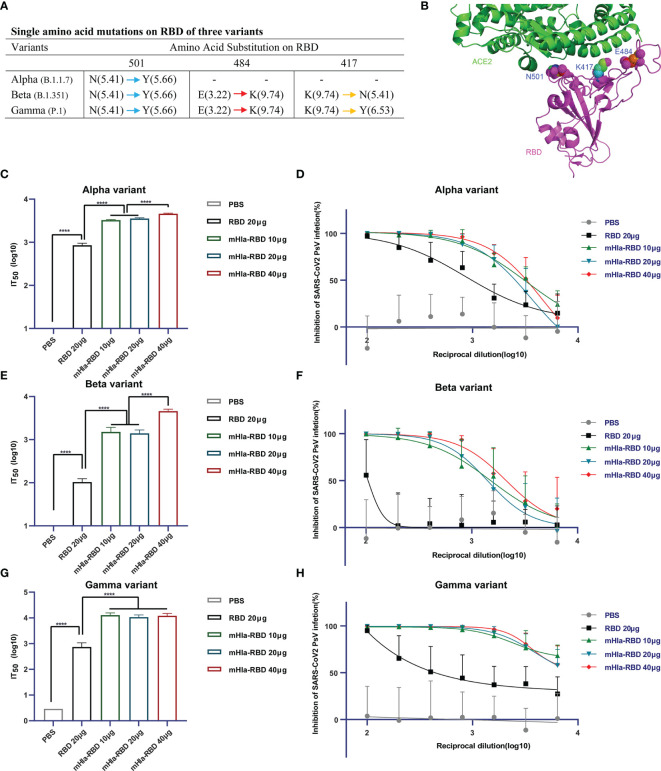
Cross-neutralization efficacy. **(A)** Details of key amino acid mutations in the RBD of three variants (N, asparagine; Y, tyrosine; E, glutamic acid; K, lysine). Values in brackets present the isoelectric point (pI) of amino acids. Red arrow means a significant change in pI. Yellow arrow means middle extent of change in pI. Blue arrow means minor change in pI. **(B)** Structural presentation of key amino acid mutations in the RBD of three variants (PDB: 6M0J). **(C)** Alpha-variant pseudovirus neutralization assay. Two-tailed unpaired Student’s t-test, ****p <0.0001. **(D)** Inhibition of alpha-variant pseudovirus infection by serial dilution of sera from immunized Balb/c mice. **(E)** Beta-variant pseudovirus neutralization assay. Two-tailed unpaired Student’s t-test, ****p <0.0001. **(F)** Inhibition of beta-variant pseudovirus infection by serial dilution of sera from immunized Balb/c mice. **(G)** Gamma-variant pseudovirus neutralization assay. Two-tailed unpaired Student’s t-test, ****p <0.0001. **(H)** Inhibition of gamma-variant pseudovirus infection by serial dilution of sera from immunized Balb/c mice.

SARS-CoV-2 keeps evolving by high-frequency mutation, enabling the virus to evade current vaccines and host immune systems. Some of these mutations may have allowed the virus to escape from neutralizing antibodies; thus, a successful vaccine should offer protection against different SARS-CoV-2 variants. To characterize the efficacy of neutralizing antibodies elicited by mHla-RBD immunization, serum samples were further tested for their neutralizing activities using pseudotyped viruses of different SARS-CoV-2 variants, including the U.K. variant B.1.1.7 (Alpha), South African variant B.1.351 (Beta), and Brazil variant P.1 (Gamma). The single amino acid mutation in the RBD of the alpha variant is N501Y. In addition to N501Y, beta and gamma variants harbor E484K, and K417 (N for beta; Y for gamma) mutations. Among these mutations, the E484K mutation results in the most significant local surface charge alteration compared to N501Y, K417N, and K417Y (**Figure 6A**), providing a plausible explanation that E484K is primarily responsible for the reduction of approved vaccine efficacy (30). Importantly, structure analysis using the RBD-ACE2 complex revealed that N501, E484, and K417 are all located at the interface between hACE2 and RBD, highlighting the importance of these residues in interaction with host cells and virus infection (**Figure 6B**).

The basal infection levels of the wild-type and alpha variant pseudoviruses were partially different (**Figures 5E**, **S1**). The normalized IT_50_ of sera from mice immunized with RBD or different dosages of mHla-RBD against wild-type pseudovirus was similar to that against alpha variant pseudovirus (**Figures 5C**, **6C**). For alpha variant pseudovirus infection, the difference in IT50 between sera immunized with RBD and mHla-RBD ranged from 3.1- to 4.3-fold (**Figures 6C, D**). However, a significant decline was observed in the inhibition of beta and gamma variant pseudovirus infection by sera immunized with RBD. This may be due to the loss of function of neutralizing antibodies recognizing E484-containing or/and K417-containing epitopes (**Figures 6E–H**). Surprisingly, sera immunized with mHla-RBD retained the majority of the ability to inhibit beta variant pseudovirus infection, and the ratio of IT_50_ between RBD and mHla-RBD was increased more than 10-fold (**Figures 6E, F**). Moreover, immunization with mHla-RBD exerted better inhibition of gamma variant pseudovirus infection than immunization with the wild-type virus (**Figures 5C**, **6G, H**). Fluorescence images intuitively showed the difference described above (**Supplementary Figure 4** to **6**). In conclusion, mHla-RBD is an excellent vaccine candidate against three prevalent variants containing RBD mutants.

## Discussion

Vaccines eliciting protective immune responses are critical to combat the current COVID-19 pandemic (31). To date, four types of SARS-CoV-2 vaccines have been approved for clinical administration, including mRNA vaccines, inactivated virus vaccines, protein subunit vaccines, and viral-vectored vaccines. The S protein was confirmed to be the most potent antigen candidate for SARS-CoV-2 vaccine development, mainly due to its critical and irreplaceable roles during the pathogenesis of the virus. Since the full-length S protein, especially the NTD of the S1 subunit, was reported to have the potential to induce antibodies involved in antibody-dependent enhancement (ADE) of disease (32, 33), concerns were raised regarding the safety of the full-length S protein as an antigen. Thus, RBD, which limits the immune response by interfering with receptor binding, attracts increasing attention for vaccine development (13). Several SARS-CoV-2 vaccines developed based on RBD have recently entered clinical trials (14, 34–36). A subunit vaccine comprised of two tandem repeats of RBD *via* a disulfide was approved by CFDA is now widely used in China (13).

However, RBD-based subunit vaccines also face some major challenges. First, the wild-type SARS-CoV-2 RBD exists as a monomer in solution, the molecular weight of which is approximately 32 kDa; thus, immunity with the RBD monomer alone cannot induce sufficient neutralizing antibodies due to poor immunogenicity (13). Second, the expression yield of RBD is relatively low and is not suitable for scaled-up for the production of large amounts of proteins (14). Third, RBD exhibits poor solubility and tends to aggregate in solutions, which may impact the conformation and immunogenicity when used as an antigen (14). As the immunogenicity is closely correlated with the molecular weight of the antigen, we introduced a carrier protein-aided assembly strategy in the current study. The designed antigen mHla-RBD formed a heptamer in solution, with a molecular weight of approximately 450 kDa, which greatly increased the size of the RBD, resulting in improved immunogenicity compared to the RBD monomer. Furthermore, the yield of the mHla-RBD is approximately 50 mg per liter culture when expressed in HEK-293F cells, which is almost 2 times higher than that of RBD (about 28 mg per liter culture), and the solubility of RBD is also improved when fusion expressed with mHla, making it a promising candidate antigen for vaccine development.

In addition to increasing the antigen size, some other mechanisms may also contribute to the mHla-mediated improvement of immunogenicity. First, ADAM10, which is highly expressed on monocytes and macrophages (37), is reported as a high-affinity host cell receptor for Hla (38). Our previous research indicated that fusion expression of an Hla mutant, termed Hla_H35L_, with protein antigens efficiently enhanced antigen uptake due to monocyte- and macrophage-dependent macropinocytosis (39). In this study, mHla fusion may also increase antigen uptake by antigen-presenting cells. Furthermore, the oligomerization of the RBD may cross-link BCRs on B cells for enhanced stimulation (13). However, further studies are required to investigate these mechanisms in detail.

In this study, the titers of RBD-specific IgG in the serum of mHla-RBD-immunized mice were significantly higher than those in RBD-immunized mice, which indicated that a higher humoral immune response was induced. Obviously, higher titers of neutralizing antibodies were induced upon mHla-RBD immunization, as indicated by the NT50 and IT50 values, which were significantly higher than those immunized with the same amount of RBD monomer (20µg). Furthermore, an IgG subtype assay revealed elevated levels of IgG1 in mHla-RBD-immunized mice, suggesting that mHla fusion efficiently enhances the Th2 immune response. This was further confirmed by ELISPOT assay, which showed that more IL-4 secreted cells were detected in spleen cells isolated from mHla-RBD immunized mice. Several studies have demonstrated that a balanced humoral and Th1 immune response is required for protection against COVID-19. Our results showed that immunization with both RBD and mHla-RBD induced IFN-γ-secreting cells compared with PBS. However, no difference was observed in different antigen-immunized groups, consistent with our previous study on IC43 (20). These results clearly demonstrated that mHla is efficient in enhancing the humoral immune response but not the cellular immune response.

An increasing number of mutations have been identified and reported in the S protein during the past year especially in RBD (40). Some of these mutations, such as K417N, L452R, E484K and N501Y, have been reported to largely reduce the efficacy of certain approved vaccines and increase the infection and pathogenicity of the virus (41–45). Thus, concerns have been raised regarding the efficacy of developed vaccines to SARS-CoV-2 variants. To our knowledge, there are two strategies to solve this problem. The first is to design new antigens harboring these mutations, and the other is to remarkably increase the titer of antibodies, especially neutralizing antibodies, toward the wild-type antigen. As an RNA virus, the genome of SARS-CoV-2 is not stable, and there is no doubt that an increasing number of mutations will occur and be identified in the coming future. The problem is that we cannot predict these mutations at present. Thus, improving the immunogenicity of RBD is a superior method for vaccine development and optimization. In the current study, the fusion of RBD with mHla resulted in improved humoral immunity. Thus, we tested the neutralization activity of serum from mHla-RBD-immunized mice using three pseudovirus variants (alpha, beta and gamma variants). Obviously, these mutations greatly enhanced the infection of host cells by the virus, as indicated by the pseudovirus infection assay. In addition, pseudovirus neutralization assays indicated that the IT50 values for variants showed no significant difference compared with wild-type virus. Furthermore, for a given variant, mHla-RBD-immunized serum showed significantly higher neutralizing capacity than RBD monomer-immunized serum. These results indicated that mHla-RBD is superior to RBD as a vaccine candidate and can provide broad-spectrum protective efficacy against different SARS-CoV-2 variants, and we will further evaluate it in animal experiments.

In conclusion, we obtained the heptamer of the SARS-CoV-2 RBD by fusion with mHla, and oligomerization of the RBD resulted in elevated levels of neutralizing antibodies, Th2 immune response and pseudovirus neutralization activity. Furthermore, conformational epitopes are predominant in RBD-mediated protection. Moreover, neutralizing antibodies induced by mHla-RBD showed broad-spectrum neutralizing activity toward different SARS-CoV-2 variants. Together with IC43, our findings demonstrated that mHla fusion may serve as a universal strategy for antigen design in developing subunit vaccines, and mHla-RBD is promising for further vaccine development.

## Data Availability Statement

The original contributions presented in the study are included in the article/**Supplementary Material**. Further inquiries can be directed to the corresponding authors.

## Ethics Statement

Animal care and use protocols were performed according to the Regulations for the Administration of Affairs Concerning Experimental Animals approved by the State Council of People’s Republic of China. All animal experiments in this study were approved by the Animal Ethical and Experimental Committee of the Third Military Medical University (Chongqing, Permit No. 2011-04) by their rules and regulations.

## Author Contributions

JYZ, HZ, and QZ designed research. JTZ, HJ, XZ, YL, LD, FY, YY, SL, and ZC performed the experiments. ZZ, JTZ, HJ, and JYZ analyzed the data. QG and QX contributed reagents/materials/analysis tools. JTZ and JYZ wrote the paper. All authors contributed to the article and approved the submitted version.

## Funding

This work was supported by grant from the National Natural Science Foundation of China (No. 31970138 and 32170938) and a program form Chongqing Education Commission (No. KYYJ202010).

## Conflict of Interest

The authors declare that the research was conducted in the absence of any commercial or financial relationships that could be construed as a potential conflict of interest.

## Publisher’s Note

All claims expressed in this article are solely those of the authors and do not necessarily represent those of their affiliated organizations, or those of the publisher, the editors and the reviewers. Any product that may be evaluated in this article, or claim that may be made by its manufacturer, is not guaranteed or endorsed by the publisher.

## Supplementary Material

Supplementary Figure 1 Determine of the titers of anti-mHla antibodies. One week after the third immunization (day 35), mHla-specific immunoglobulin gamma (IgG) antibody titers **(A)** and different dilutions of IgG **(B)** were detected by ELISA. One-way ANOVA with Tukey’s multiple comparisons, *****p <*0.0001.

Supplementary Figure 2 Hemolytic activity assay. Hemolysis activity was determined by measuring hemoglobin release of rabbit erythrocytes treated with indicated concentration of wild type Hla, mHla-RBD, 1% Triton X-100 and PBS was used as positive and negative control.

Supplementary Figure 3 Oligomeric state of RBD determined by size-elution chromatography.

Supplementary Figure 4 Presentative images of alpha-variant pseudovirus infection blocked by serial dilution of sera from immunized Balb/c mice.

Supplementary Figure 5 Presentative images of beta-variant pseudovirus infection blocked by serial dilution of sera from immunized Balb/c mice.

Supplementary Figure 6 Presentative images of gamma-variant pseudovirus infection blocked by serial dilution of sera from immunized Balb/c mice.
