# Supplementary figures and images for: α-Hemolysin-Aided Oligomerization of the Spike Protein RBD Resulted in Improved Immunogenicity and Neutralization Against SARS-CoV-2 Variants
Source: Front Immunol. 2021 Sep 24;12:757691. doi: 10.3389/fimmu.2021.757691 (PMC8497984; doi:10.3389/fimmu.2021.757691)

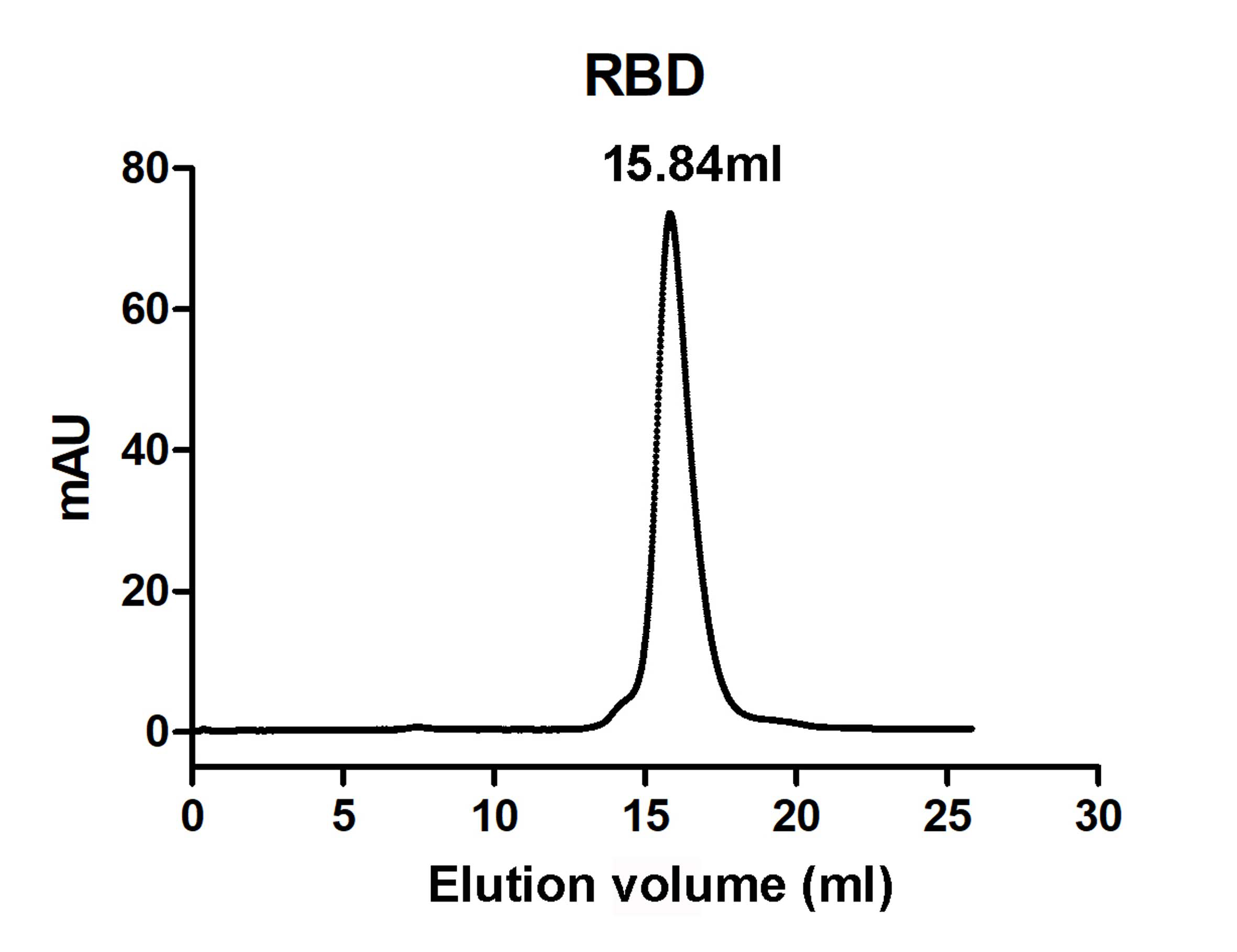

Supplement: Supplementary Figure 1 — Determine of the titers of anti-mHla antibodies. One week after the third immunization (day 35), mHla-specific immunoglobulin gamma (IgG) antibody titers (A) and different dilutions of IgG (B) were detected by ELISA. One-way ANOVA with Tukey’s multiple comparisons, ****p <0.0001. [file Image_1.tif]

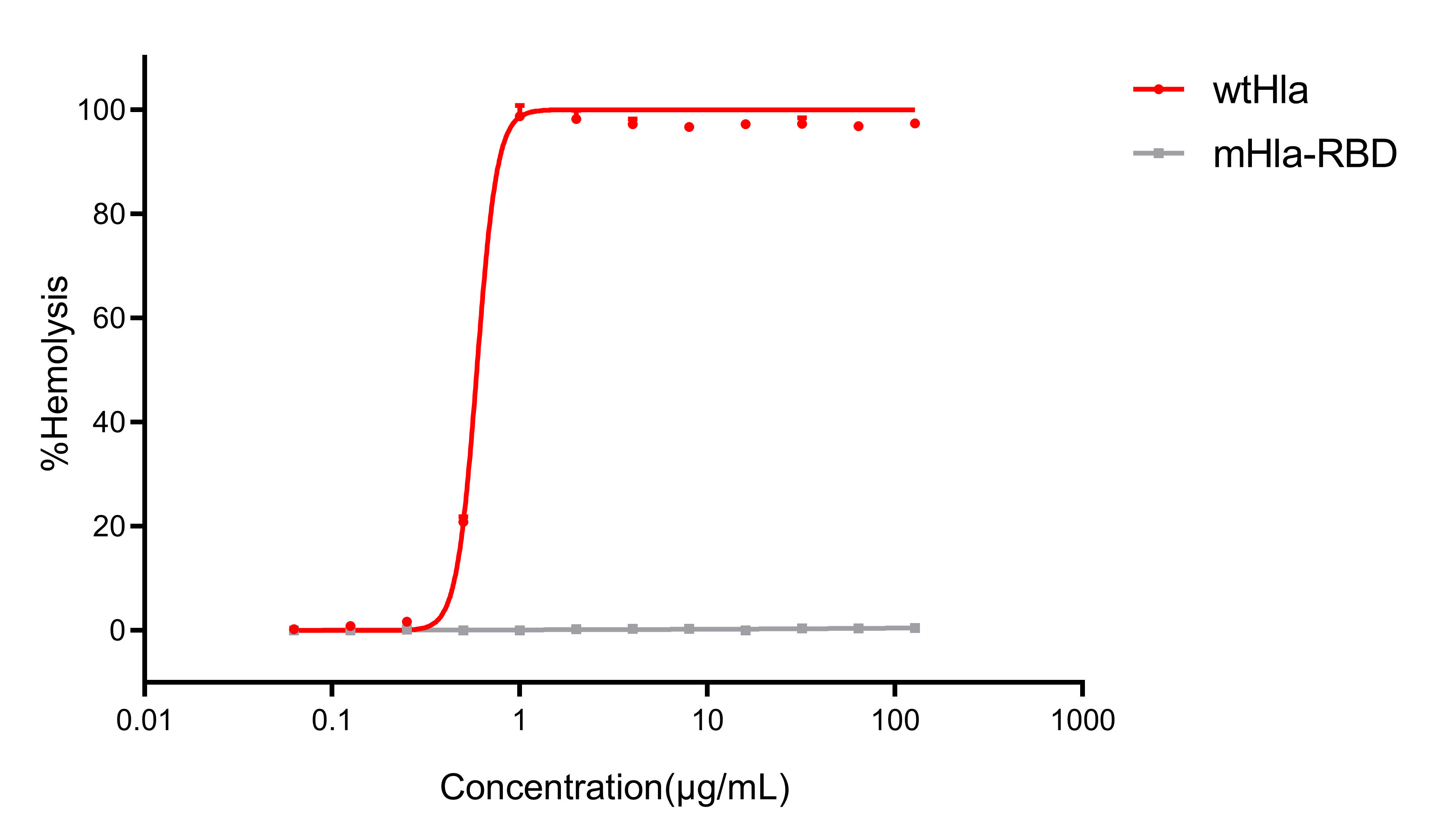

Supplement: Supplementary Figure 2 — Hemolytic activity assay. Hemolysis activity was determined by measuring hemoglobin release of rabbit erythrocytes treated with indicated concentration of wild type Hla, mHla-RBD, 1% Triton X-100 and PBS was used as positive and negative control. [file Image_2.tif]

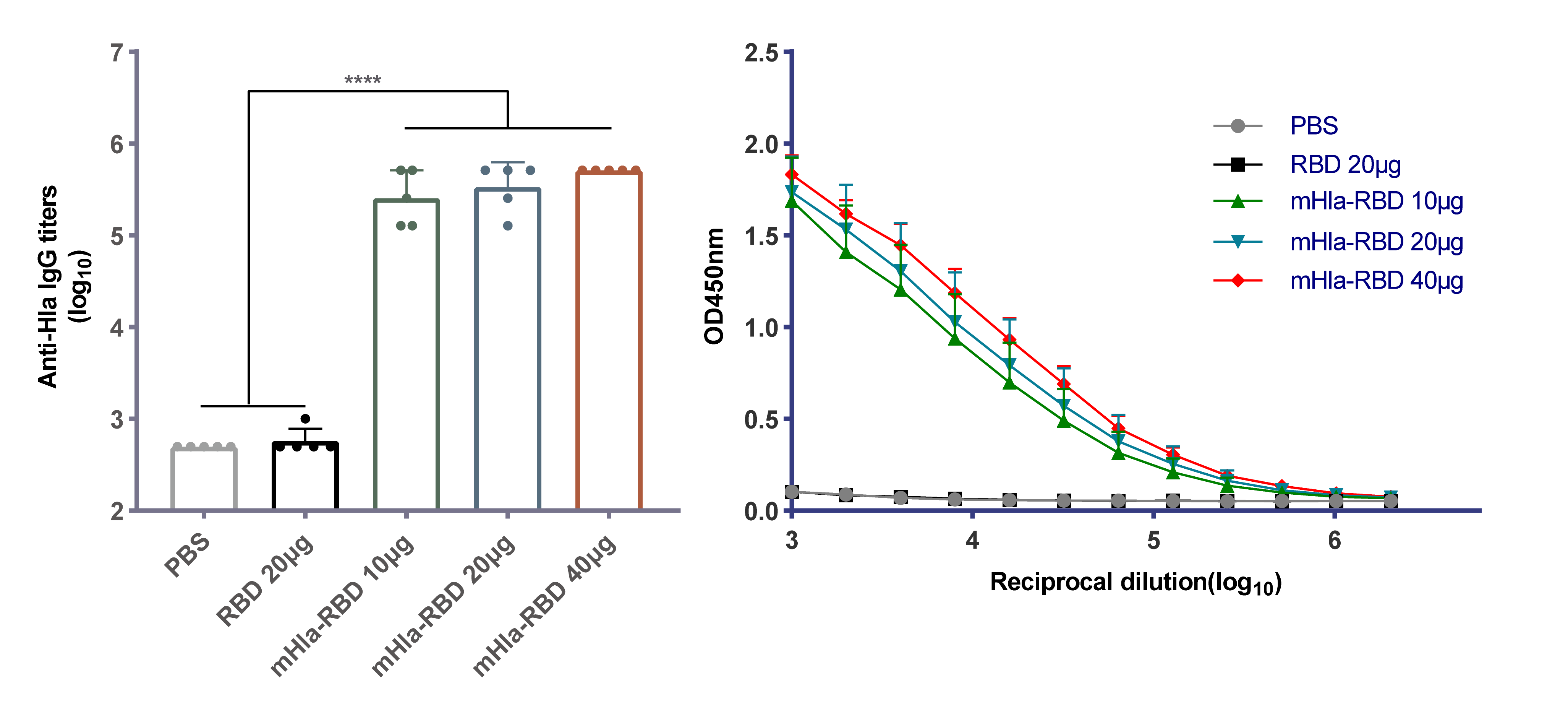

Supplement: Supplementary Figure 3 — Oligomeric state of RBD determined by size-elution chromatography. [file Image_3.tif]

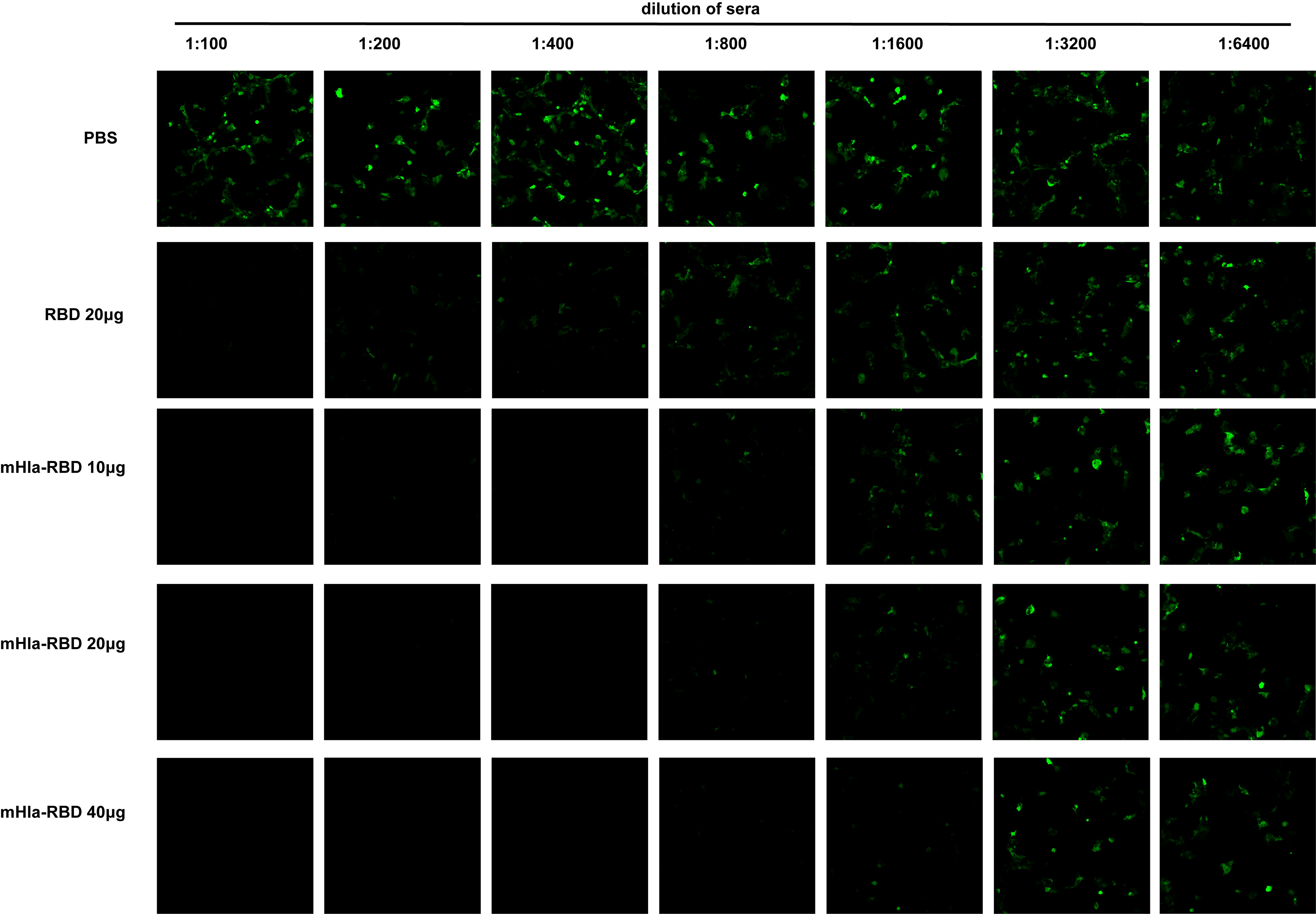

Supplement: Supplementary Figure 4 — Presentative images of alpha-variant pseudovirus infection blocked by serial dilution of sera from immunized Balb/c mice. [file Image_4.tif]

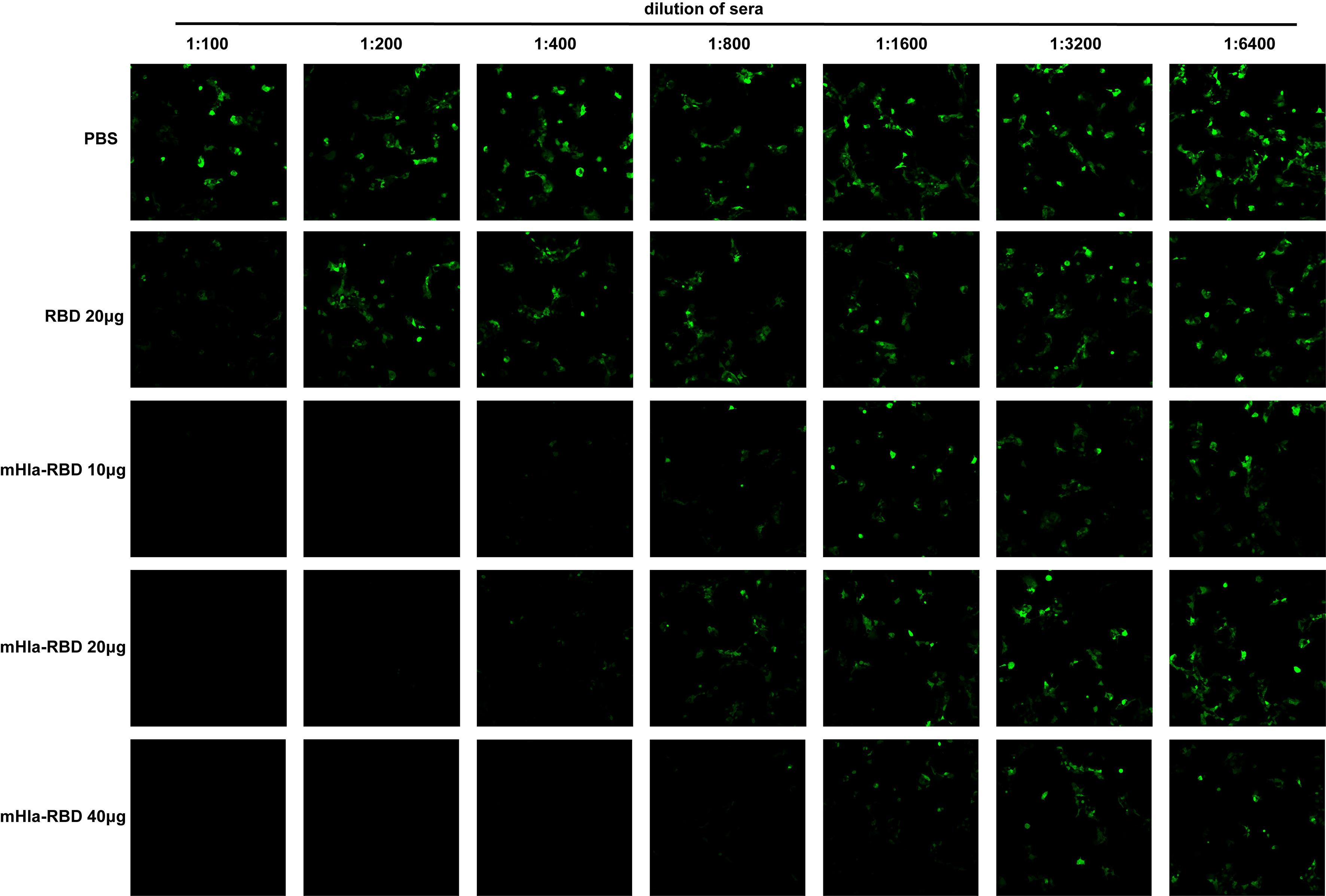

Supplement: Supplementary Figure 5 — Presentative images of beta-variant pseudovirus infection blocked by serial dilution of sera from immunized Balb/c mice. [file Image_5.tif]

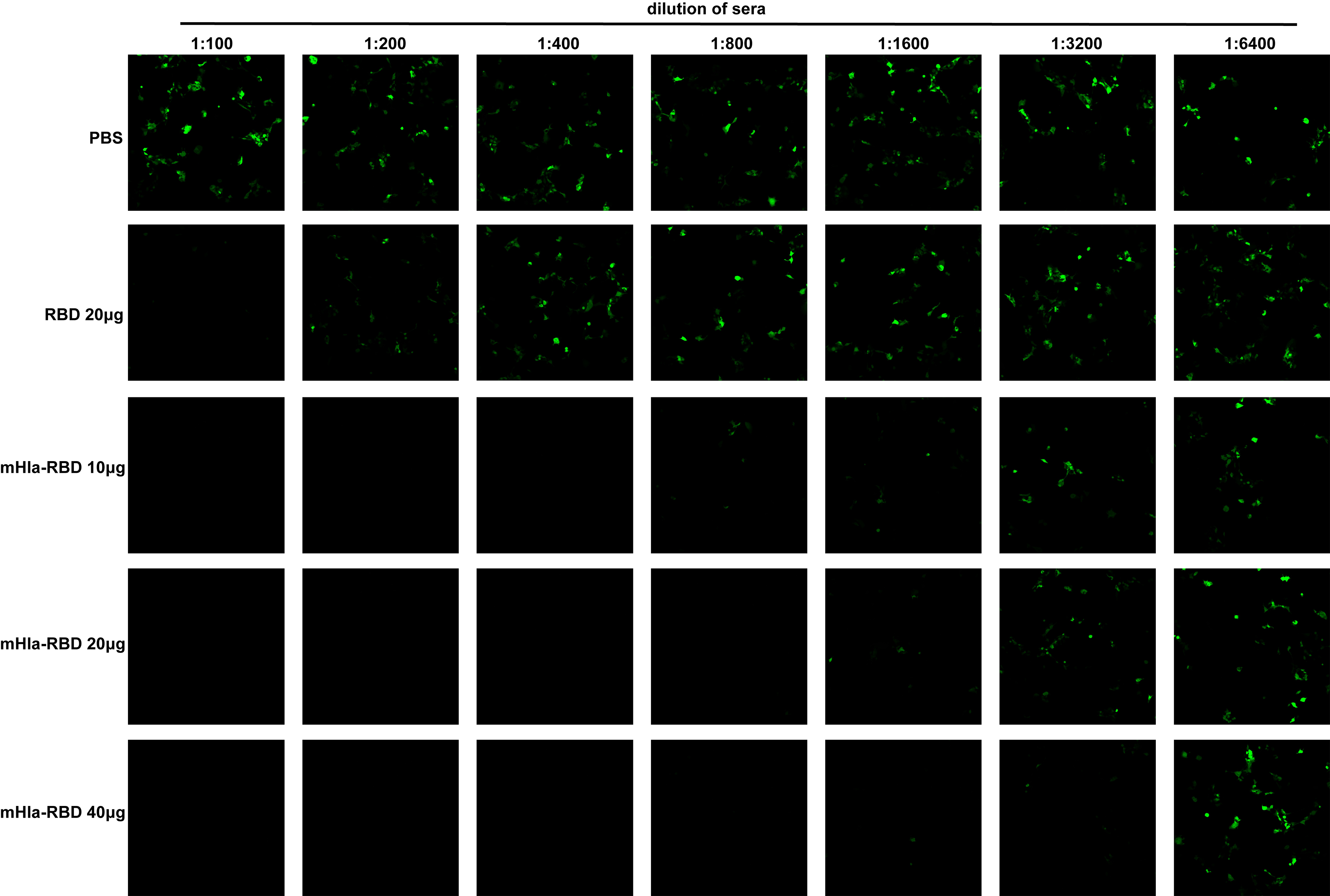

Supplement: Supplementary Figure 6 — Presentative images of gamma-variant pseudovirus infection blocked by serial dilution of sera from immunized Balb/c mice. [file Image_6.tif]
